# Prior Carriage Predicts Intensive Care Unit Infections Caused by Extended-Spectrum Beta-Lactamase–Producing Enterobacteriaceae

**DOI:** 10.4269/ajtmh.20-1436

**Published:** 2022-01-10

**Authors:** Hatem Kallel, Stephanie Houcke, Dabor Resiere, Thibault Court, Cesar Roncin, Mathieu Raad, Flaubert Nkontcho, Magalie Demar, Jean Pujo, Didier Hommel, Felix Djossou

**Affiliations:** ^1^ Intensive Care Unit, Cayenne General Hospital, Cayenne, French Guiana;; ^2^ Tropical Biome and Immunophysiopathology (TBIP), Universite de Guyane, Cayenne, French Guiana;; ^3^ Univ. de Lille, CNRS, Inserm, Institut Pasteur de Lille, Lille, France;; ^4^ Intensive Care Unit, Martinique University Hospital, Fort de France, Martinique;; ^5^ Pharmacy Department, Cayenne General Hospital, Cayenne, French Guiana;; ^6^ Laboratory of Microbiology, Cayenne General Hospital, Cayenne, French Guiana;; ^7^ Emergency Department, Cayenne General Hospital, Cayenne, French Guiana;; ^8^ Tropical and Infectious Diseases Department, Cayenne General Hospital, Cayenne, French Guiana

## Abstract

Intensive care unit–acquired infection (ICU-AI) and extended-spectrum beta-lactamase–producing *Enterobacteriaceae* (ESBL-PE) carriage are a major concern worldwide. Our objective was to investigate the impact of ESBL-PE carriage on ICU-AI. Our study was prospective, observational, and noninterventional. It was conducted over a 5-year period (Jan 2013–Dec 2017) in the medical-surgical intensive care unit of the Cayenne General Hospital (French Amazonia). During the study period, 1,340 patients were included, 271 (20.2%) developed ICU-AI, and 16.2% of these were caused by ESBL-PE. The main sites of ICU-AI were ventilator-associated pneumonia (35.8%) and primary bloodstream infection (29.8%). The main responsible microorganisms were *Staphylococcus aureus*, *Pseudomonas aeruginosa*,* Klebsiella pneumoniae* (ESBL-P in 35.8% of isolates), and *Enterobacter cloacae* (ESBL-P in 29.8% of isolates). Prior ESBL-PE carriage was diagnosed in 27.6% of patients with ICU-AI. In multivariable analysis, the sole factor associated with ESBL-PE as the responsible organism of ICU-AI was ESBL-PE carriage before ICU-AI (*P* < 0.001; odds ratio: 7.9 95% CI: 3.4-18.9). ESBL-PE carriers (74 patients) developed ICU-AI which was caused by ESBL-PE in 32 cases (43.2%). This proportion of patients carrying ESBL-PE who developed ICU-AI to the same microorganism was 51.2% in ESBL-P *K. pneumoniae*, 5.6% in ESBL-P *Escherichia coli*, and 40% in ESBL-P *Enterobacter *spp. NPV of ESBL-PE carriage to predict ICU-AI caused by ESBL-PE was above 94% and PPV was above 43%. Carriage of ESBL-P *K pneumoniae* and *Enterobacter *spp. is a strong predictor of ICU-AI caused by these two microorganisms.

## INTRODUCTION

Intensive care unit acquired infection (ICU-AI) is a major concern worldwide.^1–3^ The main responsible microorganisms are gram-negative bacteria. Among them, extended-spectrum beta-lactamase–producing *Enterobacteriaceae* (ESBL-PE) are increasingly isolated. Infections caused by ESBL-PE are associated with high ICU-mortality rates, and increased morbidity and healthcare costs.^1^ Also, because they hydrolyze penicillins, cephalosporins, and aztreonam, antibiotic options in the treatment of ESBL-PE are extremely limited.

In France, the prevalence of ESBL-PE carriage at admission to ICU varies from 3.8% to 14.2% and the acquisition rate during ICU stay varies from 1.7% to 13.2%.^4^ In south and Latin America the prevalence of ESBL-PE is among the highest worldwide.^5^ Available data from south America showed that up to 32% of *Escherichia coli *and up to 58% of *Klebsiella pneumoniae *isolates are ESBL producers. In Latin America, the commonest pathogens isolated in ICU-AI were ESBL-P *K. pneumoniae *and* E. coli* (30%).^6^ In Brazil, *K. pneumoniae* isolates from ICUs were ESBL producers in 59.2% of cases, followed by *Enterobacter *spp. (19.5%) and *E. coli* (14.6%).^7^ However, few data are available from the Amazon region.^5,8^ For this, screening for ESBL-PE is a common practice.^4^ It aims to predict related ICU-AI, and to guide empiric antibiotic therapy. However, the efficacy of screening for ESBL-PE colonization in the ICU is questioned when its prevalence is low.^9,10^

ICUs present a specific setting in which HAIs are acquired at a higher rate and exhibit higher mortality. In a systematic review, the pooled incidence of ICU-acquired sepsis was 44.8 cases per 1,000 ICU patients with a mortality rate accounting for 44.7%.^3^ In a worldwide study of patents hospitalized in ICU,^1^ 22% of patients had ICU-AI that was caused by Gram-negative microorganisms in 67% of cases. In this study, ICU-AI was independently associated to a higher risk of mortality compared with community-acquired infection. Also, ICU-AI caused by antibiotic-resistant microorganisms was independently associated with a higher risk of death compared with infection caused by antibiotic-susceptible microorganisms.^1^

The objectives of our study were to quantify ESBL-PE carriage in patients with ICU-AI and to investigate whether carriage of ESBL-PE had an impact on ICU-AI.

## MATERIALS AND METHODS

### Setting and patients.

Our study is prospective, observational, and noninterventional. It was conducted over 5 years’ period (January 2013–December 2017) in the medical-surgical intensive care unit of the Cayenne General Hospital, the only ICU in the region.^11^ It comprises 13 beds (nine single and two double-bed rooms) with a 1:2.5 nurse-to-patient ratio. All patients have dedicated equipment for care and monitoring. Hand hygiene is based on alcohol hand rub (at room entrance and exit and between each distinct procedure of care), and the use of single-use gloves and gowns in case of close contact with patients and potential exposure to body fluids during nursing. We included all patients with a first ICU admission during the same hospitalization with a stay of more than 2 calendar days. Patients hospitalized in 2012 and present in the unit on January 1, 2013 were considered as admitted the January 1, 2013. Patients readmitted during the same hospital stay were excluded from analysis.

ESBL-PE carriage was routinely screened using rectal swabbing at ICU admission and weekly afterward during the ICU stay. ESBL production was confirmed by the double-disk diffusion method using ceftazidime or cefotaxime with clavulanic acid.^12^ *Enterobacter *spp. included *E. cloacae*, *Klebsiella aerogenes*, and *E. asburiae*. Contact precautions were used for patients carrying ESBL-PE according to the French society for hospital hygiene recommendations.^13^

### Data collection.

Data of all admitted patients were prospectively collected and a detailed clinical profile was established for each patient.

The following data were collected: demographic characteristics including sex, age, type of admission, Simplified Acute Physiology Score (SAPS II),^14^ organ failure based on Sepsis-related Organ Failure Assessment (SOFA) score (defined as an acute change in total SOFA score ≥ 2 points),^2^ the main reason for admission, hospitalization and exposure to at least one dose antibiotics in the previous 12, 6, or 3 months of admission, presence of underlying diseases, exposure to central venous or arterial catheterization, mechanical ventilation, renal replacement therapy, and antibiotics during hospitalization in ICU, prior exposure to antibiotics (administration of at least one dose antibiotic during the hospitalization prior to ICU-AI), ESBL-PE carriage, infection (defined according to the definitions of the International Sepsis Forum^15^), primary bloodstream infection (BSI) was defined as a BSI without an identified source, length of ICU stay, and outcome at discharge from ICU. Microorganisms causing ICU-AI are presented according to their resistance profile. They are divided into wild strain, resistant strain, and ESBL-PE. ESBL-PE carriage was defined as the isolation of ESBL-PE from a surveillance or clinical sample. Resistant strain was defined as the resistance of microorganism to at least one beta-lactam antibiotic to which it is naturally sensitive (i.e., *Staphylococcus *spp. resistant to methicillin, Enterobacteriaceae resistant to cefotaxime, *P. aeruginosa* resistant to ceftazidime, *Acinetobacter* spp. resistant to ceftazidime). Patients with ESBL-PE isolated within 48 hours of ICU admission were considered to be colonized upon admission. ESBL-PE isolated 48 hours after admission in patients with previous negative specimens were considered as ICU acquired.^16^ Only the first episode of ICU-AI was included in the analysis, whereas infections of more than one site in the same patient were reported as independent events unless the same pathogen was isolated concurrently.

Our study was observational noninterventional and patient management falls within routine care of ICU patients. Individual patient consent was not required according to French law regarding research conforming to the norm MR-003 (JORF no. 0160 du 13 juillet 2018. texte no. 109). Our database has been registered at the Commission National de l’Informatique et des Libertés (registration no. 2209669), in compliance with French law on electronic data sources.

### Statistical analysis.

Data were described using the median and interquartile ranges (IQRs) for continuous variables and proportions (%) for categorical variables.

Initial bivariate statistical comparisons were conducted using the χ^2^ or Fisher’s exact test for categorical data and the independent-samples Student’s *t*-test for continuous data. To identify patients’ characteristics associated with ICU-AI caused by ESBL-PE, we used multivariable logistic regression with a backward procedure. Nonredundant variables selected by bivariate analysis (*P* ≤ 0.05) and considered clinically relevant were entered into a logistic regression model (i.e., gender; medical category at admission; emergent surgery; antibiotic (ATB) in the past 3, 6, or 12 months; hospitalization in the past 6 or 12 months; cancer, chronic renal failure; acute renal failure; exposure to amoxicillin clavulanate; aminoglycosides; piperacillin tazobactam before ICU-AI, ESBL-PE carriage before ICU-AI; ESBL-P *Enterobacter *spp. carriage before ICU-AI; ESBL-P K *pneumoniae* carriage before ICU-AI). Results are expressed as odds ratios (OR) with their 95% confidence intervals (CI). A *P* value ≤ 0.05 was considered statistically significant.

We calculated the sensitivity, specificity, positive (PPV) and negative predictive values (NPV), Youden test, and the Q coefficient of Yule to assess the diagnostic value of ESBL-PE carriage in predicting ESBL-PE infection.

All statistical analyses were carried out with Excel (2010 Microsoft Corporation, Redmond, WA) and IBM SPSS Statistics for Windows, version 24 (IBM Corp., Armonk, NY).

## RESULTS

**Figure 1.**
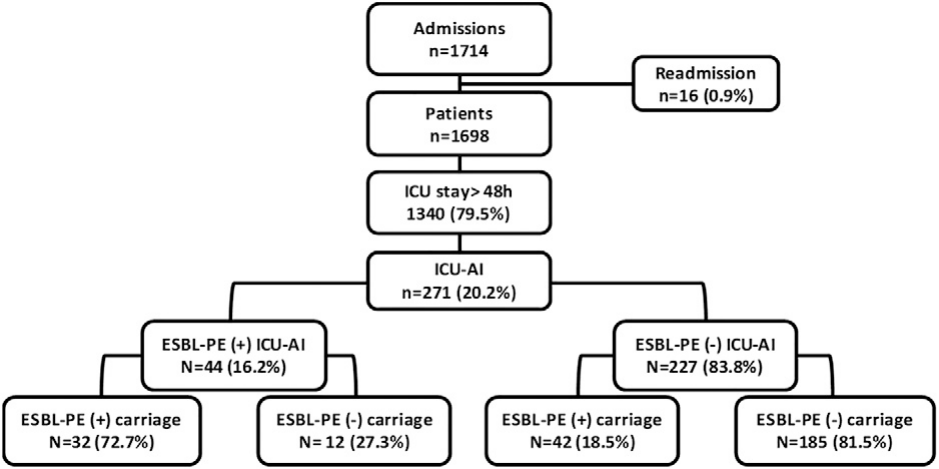
The flow-chart of the study.

During the study period, 1,698 patients were admitted to our ICU. Seventeen patients were readmitted, resulting in 1,715 admissions. The mean number of admissions varies from 316 to 380 admissions per year, and the occupancy rate per month was 84% ± 14% (IQR: 53–116). It was greater than 80% in 36 months (60% of the study period). Among admissions, 1,340 patients had an ICU length of stay (LOS) of more than 2 calendar days and were included in our study. During ICU stay, 271 patients (20.2%) developed an infection, and 44 of them (16.2%) were caused by ESBL-PE. In patients with ICU-AI, prior ESBL-PE carriage was recorded in 74 cases (27.3%). Figure 1 shows the distribution of our patients according to the occurrence of ICU-AI and to ESBL-PE carriage.

### The study population.

The median age of our patients was 45 years (IQR: 29-60) and 61.7% of them were men. Comorbidities were recorded in 46.3% of patients with hypertension, immunosuppression, and diabetes mellitus as the most common (29.9%, 15.7%, and 15.6% respectively). The main reasons for admission to ICU were trauma, respiratory failure, and coma (Table 1). Infection at admission was recorded in 48% of cases (643/1,340) and associated BSI was recorded in 14.4% of them (93/643). Antibiotics were prescribed in 66.3% of patients at admission to ICU. Epidemiological and clinical characteristics of all patients at admission to ICU are reported in Table 2.

**Table 1:** Primary reasons of admission to ICU

		ICU-acquired infection	
Reason for admission	All patients	ESBL-PE	Non-ESBL-PE
Trauma	297 (22.2%)	4 (9.1%)	81 (35.7%)
Respiratory failure	279 (20.8%)	12 (27.3%)	32 (14.1%)
Coma	184 (13.7%)	7 (15.9%)	38 (16.7%)
Sepsis	136 (10.1%)	5 (11.4%)	20 (8.8%)
Shock	114 (8.5%)	8 (18.2%)	14 (6.2%)
Monitoring	65 (4.9%)	1 (2.3%)	6 (2.6%)
Envenoming	49 (3.7%)	0 (0%)	3 (1.3%)
Metabolic disorders	36 (2.7%)	0 (0%)	4 (1.8%)
Cardiac arrest	35 (2.6%)	3 (6.8%)	12 (5.3%)
Renal failure	29 (2.2%)	2 (4.5%)	2 (0.9%)
Status epilepticus	27 (2%)	1 (2.3%)	4 (1.8%)
Intoxication	24 (1.8%)	0 (0%)	5 (2.2%)
Pregnancy complications	19 (1.4%)	0 (0%)	0 (0%)
Hepatic disorders	19 (1.4%)	1 (2.3%)	0 (0%)
Burn	13 (1%)	0 (0%)	3 (1.3%)
End of life	5 (0.4%)	0 (0%)	2 (0.9%)
Hanging	5 (0.4%)	0 (0%)	1 (0.4%)
Multiorgan failure	4 (0.3%)	0 (0%)	0 (0%)
Total	1,340 (100%)	44 (100%)	227 (100%)

ESBL-PE = extended-spectrum beta-lactamase–producing Enterobacteriaceae; ICU = intensive care unit.

**Table 2:** Epidemiological and clinical characteristics of all patients at admission to ICU

Variable	All patients, *N* = 1,340	ICU-acquired infection		*P*
		ESBL-PE, *N* = 44	Non ESBL-PE, *N* = 227	
Age, years	45 (29–60)	47 (32–62)	47 (31–60)	0.566
Gender, male	827 (61.7%)	24 (54.5%)	159 (70%)	0.045
BMI, kg/m^2^*	24.4 (21.5–28.8)	25.4 (22–33.8)	24.7 (22–29)	0.032
SAPS, points	42 (26–57)	49 (42–74)	50 (40–60)	0.046
Length of stay in ICU, days	7 (4–15)	27 (14–42)	24 (14–41)	0.533
Death	264 (19.7%)	12 (27.3%)	57 (25.1%)	0.763
Category of admission				
Medical	902 (67.3%)	37 (84.1%)	146 (64.3%)	0.010
Emergent surgery	383 (28.6%)	7 (15.9%)	74 (32.6%)	0.027
Scheduled surgery	55 (4.1%)	0 (0%)	7 (3.1%)	0.238
**ATB during** ** the past year**	**164 (12.2%)**	**13 (29.5%)**	**18 (7.9%)**	**0.001**
ATB in the past 3 months	83 (6.2%)	8 (18.2%)	9 (4%)	0.001
ATB in the past 6 months	133 (9.9%)	9 (20.5%)	17 (7.5%)	0.008
ATB in the past year	164 (12.2%)	13 (29.5%)	18 (7.9%)	0.001
**Hospitalization during the past year**	**291 (21.7%)**	**18 (40.9%)**	**37 (16.3%)**	**0.001**
Hospitalization during the past year	150 (11.2%)	7 (15.9%)	16 (7%)	0.054
Hospitalization in the past 6 months	192 (14.3%)	10 (22.7%)	20 (8.8%)	0.007
Hospitalization in the past 3 months	291 (21.7%)	18 (40.9%)	37 (16.3%)	< 0.001
**Past medical history**	620 (46.3%)	25 (56.8%)	99 (43.6%)	0.108
Hypertension	401 (29.9%)	13 (29.5%)	69 (30.4%)	0.910
Diabetes mellitus	209 (15.6%)	10 (22.7%)	36 (15.9%)	0.267
Cancer	86 (6.4%)	5 (11.4%)	8 (3.5%)	0.026
Immunosuppression	210 (15.7%)	9 (20.5%)	26 (11.5%)	0.103
Chronic renal failure	72 (5.4%)	6 (13.6%)	9 (4%)	0.010
Chronic respiratory failure	12 (0.9%)	0 (0%)	3 (1.3%)	0.443
Sickle cell disease	12 (0.9%)	1 (2.3%)	3 (1.3%)	0.632
**Organ failure**	**2 (1–3)**	**3 (2–4)**	**2 (2–3)**	
Hemodynamic failure	586 (43.7%)	32 (72.7%)	137 (60.4%)	0.121
Respiratory failure	797 (59.5%)	34 (77.3%)	152 (67.0%)	0.177
Neurologic failure	645 (48.1%)	27 (61.4%)	153 (67.4%)	0.438
Renal failure	353 (26.3%)	20 (45.5%)	63 (27.8%)	0.020
Liver failure	161 (12.0%)	7 (15.9%)	25 (11%)	0.357
Hematologic failure	252 (18.8%)	9 (20.5%)	42 (18.5%)	0.762

ATB = antibiotic; BMI = body mass index; ESBL-PE = extended-spectrum beta-lactamase–producing Enterobacteriaceae; ICU = intensive care unit; SAPS = Simplified Acute Physiology Score.

* BMI was available in 1,029 cases (41 in the ESBL-PE and 185 in the non-ESBL-PE group).

### Therapeutic management in ICU.

During ICU stay, 64.1% of patients (859/1,340) received invasive mechanical ventilation, 10.3% (138/1,340) received renal replacement therapy, 68.7% (921/1,340) had central venous catheterization, and 63.5% (851/1,340) had arterial catheterization. Antibiotic exposure during ICU stay was recorded in 69.2% of patients (927/1,340). Therapeutic procedures and antibiotics exposure before ICU-AI are reported in Table 3.

**Table 3:** Therapeutic procedures and antibiotics exposure before ICU-AI

	All patients, *N* = 1340	ICU acquired infection		*P*
Variable		ESBL-PE, *N* = 44	Non-ESBL-PE, *N* = 227	
Mechanical ventilation	859 (64.1%)	42 (95.5%)	205 (90.3%)	0.271
Time from admission to MV, days	0 (0–0)	0 (0–0)	0 (0–0)	0.486
Duration of MV, days	6 (3–15)	19.5 (13–32)	19 (11–29)	0.813
Tracheostomy	51 (5.9%)	4 (9.5%)	31 (15.1%)	0.343
Renal Replacement Therapy	138 (10.3%)	14 (31.8%)	34 (15%)	0.007
Time from admission to RRT, days	0 (0–1)	1 (0–3)	1 (0–5)	0.563
Central Venous Catheter	921 (68.7%)	43 (97.7%)	216 (95.2%)	0.448
Duration of CVC, days	9 (5–18)	20 (14–40)	18 (13–31)	0.099
Arterial Catheter	851 (63.5%)	41 (93.2%)	202 (89%)	0.403
Duration of AC, days	7 (4–13)	16 (12–21)	14 (9–21)	0.095
Prior exposure to antibiotics	206 (76.1%)	38 (86.4%)	168 (74%)	0.079
Amoxicillin clavulanate	99 (36.5%)	7 (15.9%)	92 (40.5%)	0.002
Aminoglycosides	87 (32.1%)	24 (54.5%)	63 (27.8%)	< 0.001
Piperacillin Tazobactam	60 (22.1%)	18 (40.9%)	42 (18.5%)	0.001
3rd Generation Cephalosporins	48 (17.7%)	11 (25%)	37 (16.3%)	0.166
Carbapenems	26 (9.6%)	7 (15.9%)	19 (8.4%)	0.120
Quinolones	24 (8.9%)	7 (15.9%)	17 (7.5%)	0.072
Metronidazole	4 (1.5%)	1 (2.3%)	3 (1.3%)	0.632

AC = arterial catheter; CVC = central venous catheter; ESBL-PE = extended-spectrum beta-lactamase–producing Enterobacteriaceae; ICU-AI = intensive care unit–acquired illness; MV = mechanical ventilation; RRT = renal replacement therapy. Prior exposure to antibiotics: calculated only in patients with ICU-AI.

### ICU-AI.

During ICU stay, 271 of 1,340 patients (20.2%) developed ICU-AI. The median time from admission to ICU-AI was 8 days (IQR: 5–13 days). ICU-AI was caused by an ESBL-PE in 44 of 271 patients (16.2%). The main sites of ICU-AI were ventilator-associated pneumonia (VAP), primary BSI, and catheter-related infection (Table 4). The responsible microorganisms are reported in Table 5. They include mainly *S. aureus*, *Pseudomonas aeruginosa*,* K. pneumoniae* (ESBL-P in 36% of isolates), and *Enterobacter cloacae* (ESBL-P in 30% of isolates).

**Table 4:** The sites and associated bacteraemia of ICU-AI

	Associated bacteraemia		Total no. ICU-AI
	No	Yes	
	141	130	271
VAP	76 (52.8%)	26 (18.4%)	102 (35.8%)
Primary BSI	0 (0%)	85 (60.3%)	85 (29.8%)
Catheter related infection	29 (20.1%)	22 (15.6%)	51 (17.9%)
Urinary tract infection	12 (8.3%)	4 (2.8%)	16 (5.6%)
Pneumonia	14 (9.7%)	1 (0.7%)	15 (5.3%)
Surgical site infection	4 (2.8%)	2 (1.4%)	6 (2.1%)
Skin	3 (2.1%)	0 (0%)	3 (1.1%)
Meningitis	2 (1.4%)	0 (0%)	2 (0.7%)
Peritonitis	2 (1.4%)	0 (0%)	2 (0.7%)
Splenic abscess	1 (0.7%)	0 (0%)	1 (0.4%)
Endocarditis	0 (0%)	1 (0.7%)	1 (0.4%)
Bone	1 (0.7%)	0 (0%)	1 (0.4%)
Total	144 (100%)	141 (100%)	285 (100%)

BSI = bloodstream Infection; ICU-AI = intensive care unit–acquired illness; VAP = ventilator-associated pneumonia. Fourteen patients presented ICU-AI at two sites.

**Table 5:** The responsible microorganisms of intensive care unit–acquired illness

	Wild strain	Resistant strain		Total
Gram-positive cocci				
*Staphylococcus aureus*	40 (93%)	3 (7%)		43 (100%)
CNS	8 (50%)	5 (50%)		13 (100%)
*Streptococcus agalactiae*	2 (100%)	0 (0%)		2 (100%)
*Enterococcus faecalis*	6 (100%)	0 (0%)		6 (100%)
*Streptococcus oralis*	1 (100%)	0 (0%)		1 (100%)
Nonfermentative Gram-negative bacteria				
*Pseudomonas aeruginosa*	33 (94.3%)	2 (5.7%)		35 (100%)
*Acinetobacter baumanii*	19 (90.5%)	2 (9.5%)		21 (100%)
*Aeromonas hydrophila*	2 (100%)	0 (0%)		2 (100%)
*Acinetobacter nosocomialis*	1 (100%)	0 (0%)		1 (100%)
*Burkholderia cepacia*	4 (100%)	0 (0%)		4 (100%)
*Stenotrophomonas maltophilia* ^*^	0 (0%)	5 (100%)		5 (100%)
Enterobacteriaceae		Non ESBL-PE	ESBL-PE	
*Escherichia coli*	20 (87%)	0 (0%)	3 (13%)	23 (100%)
*Klebsiella pneumoniae*	52 (64.2%)	0 (0%)	29 (35.8%)	81 (100%)
*Enterobacter cloacae*	29 (61.7%)	4 (8.5%)	14 (29.8%)	47 (100%)
*Klebsiella aerogenes*	11 (84.6%)	1 (7.7%)	1 (7.7%)	13 (100%)
*Enterobacter asburiae*	1 (50%)	0 (0%)	1 (50%)	2 (100%)
*Morganella morganii*	1 (100%)	0 (0%)	0 (0%)	1 (100%)
*Proteus mirabilis*	4 (100%)	0 (0%)	0 (0%)	4 (100%)
*Providentia stuartii*	1 (100%)	0 (0%)	0 (0%)	1 (100%)
*Serratia marcessens*	8 (80%)	1 (10%)	1 (10%)	10 (100%)
Other bacteria				
*Neisseria meningitidis*	1 (100%)	**–**		1 (100%)
*Haemophilus influenzae*	3 (100%)	**–**		3 (100%)
*Candida *spp.				
*Candida albicans*				7 (100%)
*Candida koseri*				5 (100%)
*Candida parapsilosis*				3 (100%)
None isolated				9 (100%)

CNS = coagulase negative staphylococci; ESBL-PE = extended-spectrum beta-lactamase-producing Enterobacteriaceae.

* *S. maltophilia* is a naturally resistant nonfermentative bacteria.

### ESBL-PE carriage at admission and during ICU stay.

ESBL-PE carriage was diagnosed in 10% of patients at ICU admission and in 19.6% (of noncarriers at admission) during ICU stay. The median time from admission to ESBL-PE acquisition was 10 days (IQR: 6–16). It was shorter in patients with ICU-AI caused by ESBL-PE (*P* < 0.001). Table 6 reports ESBL-PE carriage during ICU stay and before ICU-AI.

**Table 6:** ESBL-PE carriage during ICU stay

			ICU-AI				
Variable	nb	All patients	nb	ESBL-PE	nb	Non-ESBL-PE	*P*
ESBL-PE carriage during ICU stay	1,340	370 (27.6%)	44	44 (100%)	227	109 (48%)	< 0.001
ESBL-PE carriage at admission	1,340	134 (10%)	44	11 (25%)	227	17 (7.5%)	< 0.001
ICU acquired ESBL-PE	1,206	236 (19.6%)	33	33 (100%)	210	92 (43.8%)	< 0.001
Time from admission to ESBL-PE acquisition	236	10 (6–16)	33	6 (4–8)	92	15 (10–21)	< 0.001
ESBL-P *K. pneumoniae* carriage	1,340	204 (15.2%)	44	32 (72.7%)	227	64 (28.2%)	< 0.001
ESBL-P *E. coli* carriage	1,340	123 (9.2%)	44	10 (22.7%)	227	31 (13.7%)	0.124
ESBL-P *Enterobacter *spp. carriage	1,340	111 (8.3%)	44	16 (36.4%)	227	37 (16.3%)	0.002
ESBL-PE carriage before ICU-AI	271	74 (27.3%)	44	32 (72.7%)	227	42 (18.5%)	< 0.001
ESBL-P *K. pneumoniae*	271	43 (15.9%)	44	23 (52.3%)	227	20 (8.8%)	< 0.001
ESBL-P *E. coli*	271	18 (6.6%)	44	4 (9.1%)	227	14 (6.2%)	0.476
ESBL-P *Enterobacter *spp.	271	20 (7.4%)	44	9 (20.5%)	227	11 (4.8%)	< 0.001
ICU-AI	1,340	271 (20.2%)	44	44 (100%)	227	227 (100%)	–
ICU-AI caused by ESBL-PE	271	44 (16.2%)	44	44 (100%)	227	0 (0%)	**–**
ICU-AI caused by ESBL-P *K. pneumoniae*	271	29 (10.7%)	44	29 (65.9%)	227	0 (0%)	**–**
ICU-AI caused by ESBL-P *E. coli*	271	2 (0.7%)	44	2 (4.5%)	227	0 (0%)	**–**
ICU-AI caused by ESBL-P *Enterobacter *spp.	271	16 (5.9%)	44	16 (36.4%)	227	0 (0%)	**–**

ESBL-P = ESBL producer; ESBL-PE = extended-spectrum beta-lactamase–producing Enterobacteriaceae; ICU = intensive care unit; ICU-AI = intensive care unit–acquired illness.

### Predictive factor of ICU-AI caused by ESBL-PE.

In multivariable analysis, the sole factor associated to ESBL-PE as the responsible organism of ICU-AI was ESBL-PE carriage before ICU-AI (*P* < 0.001; OR: 7.9 [3.4–18.9]).

### Value of ESBL-PE carriage to predict ICU-AI caused by ESBL-PE.

In patients with ICU-AI, 32 of the 74 ESBL-PE carriers (43%) developed infections caused by ESBL-PE. This rate was 51% in ESBL-P *K. pneumoniae*, 40% in ESBL-P *Enterobacter *spp., and 6% in ESBL-P *E. coli*. NPV of carriage of ESBL producing *Enterobacteriaceae*, *E. coli*, *K. pneumoniae*, or *Enterobacter *spp. to predict ICU-AI due to the same microorganism was above 94% in the four groups whereas the PPV was (43%, 6%, 51%, and 40% respectively). Table 7 reports the diagnostic value of ESBL-PE carriage to predict ICU-AI caused by the same microorganism.

**Table 7:** Diagnostic value of ESBL-PE carriage to predict ICU-acquired illness caused by the same microorganism

Carriage	TP	FP	TN	FN	Sn	Sp	PPV	NPV	Q	Youden
ESBL-PE	32	42	185	12	0.727	0.815	0.432	0.939	0.843	0.542
ESBL-P *E. coli*	1	17	252	1	0.050	0.937	0.056	0.996	0.874	0.437
ESBL-P *K. pneumoniae*	22	21	221	7	0.759	0.913	0.512	0.969	0.941	0.672
ESBL-P *Enterobacter *spp.	8	12	243	8	0.500	0.953	0.400	0.968	0.906	0.453

ESBL-PE = extended-spectrum beta-lactamase–producing Enterobacteriaceae; FN = false negative; FP = false positive; ICU = intensive care unit; NPV = negative predictive value; PPV = positive predictive value; Q = coefficient of Yule; Sn = sensitivity; Sp = specificity; TN = true negative; TP = true positive.

## DISCUSSION

Our study provides information about colonization and infection to ESBL-PE in ICU in the French Amazonian context. The main findings of our study are that ESBL-PE carriage in our ICU is similar to that reported in other French ICU despite the South American location of our hospital. ESBL-PE carriage is frequently associated with ICU-AI and can predict ESBL-PE as the responsible organism of ICU-AI.

ICU-Ais affect 16% to 22% of patients admitted to ICUs and are independently associated with a higher risk of mortality compared with community-acquired infection.^1^ The responsible organisms are mainly Gram-negative microorganisms that are associated with a high risk of death in case of resistance. In our study, ICU-AI was diagnosed in 20.2% of cases. The main sites of ICU-AI were VAP, primary BSI, and catheter-related infection. These findings indicate that the epidemiology of ICU-AI is similar to that reported in mainland France, Europe, and North America.^1^ This result is interesting giving the South American and Amazonian location of our hospital. It can explained by the prevention and management guidelines strategies used in our hospital, which are based on international and French standards.^11^

ESBL-PE is major concern worldwide. Surveillance networks reveal a predominance of *K. pneumoniae* in Latin America and Asia Pacific region with a lower incidence in Europe and North America.^17,18^ Indeed, in Latin America the prevalence rate of ESBL-PE is among the highest in the world reaching 51% for *K. pneumoniae* and 18% for *E. coli*.^19,20^ In our study, ESBL-PE carriage was found in 10% of patients at admission and was acquired in 19.6% during hospitalization in ICU. The main ESBL-PE isolated in the screening tests was *K. pneumoniae*. Our results are similar to those from Europe and North America and show a lower level of ESBL-PE carriage than would be predicted by the South American location of our hospital.

The impact of ESBL-PE carriage on ICU-AI is controversial. In some studies, ESBL-PE carriage was reported to be associated with a higher risk of subsequent infection in ICU patients.^4,9,21,22^ Andremont et al.^21^ found that a high-density ESBL-PE rectal carriage is a risk factor of VAP caused by ESBL-PE. Houard et al.^22^ reported that previous ESBL-PE fecal carriage is independent risk factor predicting ESBL-PE VAP (OR 23; 95% CI: 10–55%, *P* < 0.001). However, other recent studies found that the incidence of ICU-AI caused by ESBL-PE is relatively low in carriers (10–25%).^10,23–26^ In a prospective study, Razazi et al.^25^ found that in carriers, ESBL-PE cause only 10% and 27% of first and second episodes of ICU-AI, respectively. Barbier et al.^27^ found that among the 318 enrolled ESBL-PE carriers, only 7% developed infections caused by ESBL-PE. A similar result was found by Lindblom et al.,^24^ who concluded that infections caused by ESBL-PE in previously colonized patients are rare. In addition, some authors reported that switching from universal to targeted active surveillance cultures had no impact on the incidence of ICU-acquired ESBL-PE infections.^28,29^ All these findings led some authors to suspect that screening for ESBL-PE carriage is powerless in predicting subsequent infection, and it can be a driver to an overuse of carbapenems.^10^ Thus, recent studies have challenged the benefit of active surveillance cultures to detect intestinal carriage of ESBL-PE in controlling the spread of ESBL-PE in ICUs with high compliance to standard hygiene precautions and no ongoing outbreak of ESBL-PE.^9,10^ In our study, 370 patients were ESBL-PE carriers. ICU-AI was diagnosed in 20.2% of patients, and ESBL-PE carriage before ICU-AI was recorded in 27.3% of cases. ICU-AI was caused by an ESBL-PE in 16.2% of cases, and in 59.5% of ESBL-PE carriers. In addition, ESBL-PE carriage before ICU-AI was the sole independent factor associated with ICU-AI caused by ESBL-PE, and showed interesting values to predict ICU-AI caused by ESBL-PE (PPV: 43.2%, NPV: 93.9%). The highest prediction value was observed with *K. pneumoniae* and the lowest with *E. coli*. These results are concordant with other studies^4,9,21,22^ and can be explained, in part, by the high prevalence of primary BSI which are commonly caused by bacterial translocation from the digestive tract.^8^

Our study has four limitations. First, this is a single-center study. However, our unit is the sole ICU in French Guiana.^11^ For this reason, the overview of the local situation is almost exhaustive. The second limitation is that bacterial identification was only phenotypic without information on the genotypic typing of ESBL. Third, rectal swab cultures were performed on a weekly basis that cannot determine with accuracy the date of ESBL-PE acquisition. Fourth, rectal swab cultures were only qualitative. However, to the best of our knowledge, this study is the first one reporting ESBL-PE carriage and infections in the Guiana shield and in the French Territories of the Americas. Further studies are needed to explore the genotypic typing of ESBL and to search for decision-making tools for a relevant stewardship of antibiotics in patients carrying ESBL-PE.

## CONCLUSION

ESBL-PE carriage and ICU-AI are major concerns in French Guiana as in other parts of the world. The prevalence of ESBL-PE carriage is similar to that reported in mainland France ICUs despite the oversea location of our hospital. The absence of ESBLE-PE carriage has a high NPV, which would suggest ESBLE-PE is not responsible in the case of ICU-AI.
